# Medium-Chain Fatty Acids Selectively Sensitize Cancer Cells to Ferroptosis by Inducing CD36 and ACSL4

**DOI:** 10.3390/nu17050794

**Published:** 2025-02-25

**Authors:** Kai Han, Jiaxuan Li, Shutao Yin, Hongbo Hu, Chong Zhao

**Affiliations:** College of Food Science and Nutritional Engineering, China Agricultural University, No. 17 Qinghua East Road, Haidian District, Beijing 100083, China; hankai1122@cau.edu.cn (K.H.); ljx620@cau.edu.cn (J.L.); yinshutao@cau.edu.cn (S.Y.)

**Keywords:** medium-chain fatty acids, cancer, ferroptosis, CD36, ACSL4

## Abstract

Background: Inducing ferroptosis in cancer cells is a promising therapeutic strategy. It has been shown that certain types of fatty acids can induce ferroptosis in multiple types of cancer cells. Methods: Here, we employed crystal violet staining and CCK8 to assess cell viability, a Liperfluo probe and commercial kit to measure lipid peroxides, and western blotting and RNA interference to detect protein levels. Results: This study demonstrates for the first time that the medium-chain fatty acids lauric acid (LA-m), octanoic acid (OA-m), and decanoic acid (DA-m) selectively sensitize various cancer cell types to ferroptosis induced by either RSL3, a well-known inducer of ferroptosis, or linoleic acid (LA-l), a ω-6 polyunsaturated fatty acid (PUFA). Mechanistically, the ferroptosis-sensitizing effect of medium-chain fatty acids is associated with their ability to upregulate cluster of differentiation 36 (CD36) and acyl-CoA synthetase long-chain family member 4 (ACSL4) expression. Conclusions: These findings suggest that medium-chain fatty acids could be developed as novel ferroptosis sensitizers to enhance ferroptosis-based cancer therapy.

## 1. Introduction

Ferroptosis is an iron-dependent form of regulated cell death driven by excessive oxidation of polyunsaturated phospholipids on cellular membranes, and was described by Dixon et al. in 2012 [1]. Substantial evidence indicates that ferroptosis plays a role in the pathogenesis of various diseases, including cancer [2,3,4]. Ferroptosis was originally identified in cancer cells, when Dixon et al. reported that a unique iron-dependent form of non-apoptotic cell death was induced by the small molecule erastin in a number of cancer cells, including NRAS-mutant HT-1080 fibrosarcoma cells, HRAS-mutant BJeLR engineered tumor cells and KRAS-mutant Calu-1 non-small cell lung cancer cells [1]. Ferroptosis has increasingly been recognized as an important tumor-suppressive mechanism. Understanding of the role of ferroptosis in carcinogenesis offers a new therapeutic avenue for cancer management, particularly for patients with cancer types that are refractory to conventional therapies. An increasing number of studies support ferroptosis-based strategies as promising approaches for enhancing cancer treatment efficacy [5,6,7,8].

Numerous studies have shown that ferroptosis can be regulated by a number of nutrients, including fatty acids [9,10,11]. The regulatory effect of fatty acids on ferroptosis is context-dependent, mainly depending on the types of fatty acids and the types of cells. Generally, ω-6 polyunsaturated fatty acids (PUFAs), mainly arachidonic acid (AA, C20:4) and adrenic acid (AdA, C22:4), have been identified as the key substrates of lipid peroxidation during ferroptosis induction, thus promoting ferroptosis [12], whereas monounsaturated fatty acids suppress ferroptosis by displacing PUFAs from plasma membrane phospholipids which renders the plasma membrane structure resistant to lipid peroxidation and ferroptosis induction [13]. ω-3 PUFAs protect normal cells from the ferroptosis attributed to the activation of the interferon regulating factor 3 (IRF3)–solute carrier family seven member 11 (SLC7A11)–lipoxygenase 12 (ALOX12) antioxidant system [14]; however, they promote ferroptosis in certain types of cancer cells associated with the suppression of dihydrofolate reductase (DHFR) and ferroptosis suppressor protein-1 (FSP-1) [15]. Long-chain saturated fatty acids (palmitic acid) have been reported to enhance production of PUFAs, mainly AA [16], or inhibiting the Xc^−^/GSH/GPX4 antioxidant system [17,18,19], therefore facilitating ferroptosis. However, the role of medium-chain fatty acids in ferroptosis regulation remains well elusive. Using a mouse model of chronic sleep deprivation (SD), Wang et al. showed that a medium-chain triglyceride-enriched ketogenic diet (MKD) significantly improved cognitive impairment, which was correlated with suppressing neuronal cell ferroptosis [20]. More studies are clearly needed to better understand the role of medium-chain fatty acids in the regulation of ferroptosis. To this end, we investigated the effect of medium-chain fatty acids on the sensitivity of cancer and normal cells to ferroptosis. Results show that octanoic acid (OA-m), decanoic acid (DA-m), and lauric acid (LA-m) significantly increased the sensitivity of cancer cells, but not normal cells, to the ferroptosis induced by linoleic acid (LA-l), a representative of ω-6 PUFAs, and RSL3, a well-known inducer of ferroptosis which targets the GPX4 antioxidant enzyme.

## 2. Materials and Methods

### 2.1. Reagents and Antibodies

Lauric acid (LA-m) (purity ≥ 99.98%), linoleic acid (LA-l) (purity ≥ 99.92%), octanoic acid (OA-m) (purity = 99.70%), decanoic acid (DA-m) (purity ≥ 98.0%), (1S,3R)-RSL3 (RSL3, a ferroptosis inducer), liproxstatin-1 (Lip-1, a ferroptosis inhibitor), sulfosuccinimidyl oleate sodium (SSO, a CD36 inhibitor), and rosiglitazone (ROSI, a ACSL4 inhibitor) were obtained from MedChemExpress (Monmouth Junction, NJ, USA). Liperfluo (LPO) was acquired from DOJINDO (Kyushu, Japan). CD36 (#ab252922) antibody was purchased from Abcam (Cambridge, MA, USA). ACSL4 (sc-365230) was purchased from Santa Cruz Biotechnology (Santa Cruz, CA, USA). The antibodies for β-actin and GAPDH were obtained from ABclonal (Wuhan, China). The specific second antibodies for rabbit and mouse were obtained from MBL International Corporation (Beijing, China).

### 2.2. Cell Culture

All cell lines were provided by the American Type Culture Collection (ATCC). U937 human leukemia cells and A549 lung cancer cells were cultured in RPMI-1640 medium, while Lewis lung cancer (LLC) cells and 293T human embryonic kidney cells were maintained in DMEM. MRC-5 human embryonic lung cells and HK2 human proximal tubular epithelial cells were grown in an MEM medium. RINm5F rat insulinoma cells were grown in RPMI-1640 medium (without HEPES). AML12 alpha mouse liver cells were cultured in DMEM/F12 medium plus 1% ITS. All of these culture media were supplemented with 10% fetal bovine serum. The cells were incubated in a humidified atmosphere containing 5% CO_2_ at 37 °C, and until they reached a confluence of 40% to 50%. Then, the cells were exposed to an indicated complete medium containing different agents.

### 2.3. Cell Viability Assessment

*Crystal Violet Staining:* Cells (1 × 10^5^ cells/well) were embedded in a 12-well plate. Following the administration of drugs for indicated times, the culture medium was removed, and cells were fixed in 1% glutaraldehyde solution for 15 min and then stained with 0.02% crystal violet solution for 30 min. Subsequently, the stained cells were solubilized with 75% ethanol. The OD value at a wavelength of 570 nm was determined using a microplate reader (Thermo).

*Cell Counting Kit-8 (CCK-8):* Cells (1  ×  10^3^ cells/well) were seeded into 96-well plates. The next day, the cells were treated with indicated agents and cultured for another 24 h. The CCK-8 assay (Dojindo, Tokyo, Japan) was then utilized to evaluate cell proliferation rates, following the manufacturer’s instructions.

### 2.4. Lipid Peroxides Measurement

Cells (2 × 10^5^ cells/well) were plated into a 6-well plate. Following the treatments, the cells were trypsinized and subsequently washed twice with PBS. They were then incubated with 1 µM Liperfluo for 30 min at 37 °C in the dark. After incubation, the cell fluorescence intensity was analyzed by flow cytometer (Becton Dickinson, Franklin Lakes, NJ, USA). The specific excitation and emission wavelengths were 488 nm and 535 nm, respectively.

### 2.5. Measurement of Malondialdehyde (MDA)

Cells (2 × 10^5^ cells/well) were seeded in a 6-well plate. The next day, the culture medium was replaced with the indicated treatments. Then, cells were harvested using sterile PBS. The intracellular MDA levels (MDA kit: #Cat. A003-1-2) were determined following the protocols provided by Nanjing Jiancheng Bioengineering Institute (Nanjing, China).

### 2.6. Western Blotting

Cells were lysed in a mixture containing ice-cold RIPA buffer, protease, and phosphatase inhibitors (Solarbio Life Science, Beijing, China). Next, aliquots of protein samples were separated by SDS-PAGE and then transferred to a polyvinylidene fluoride (PVDF) membrane (Millipore, Billerica, MA, USA; IPVH00010). After blocking with 5% non-fat dry milk, the membrane was incubated with primary antibodies at 4 °C overnight. Afterward, the membrane was incubated with an HRP-conjugated secondary antibody for 1 h at room temperature. Protein expression was detected using enhanced chemiluminescence. The bands were quantified using Image J version 1.8.0 software, normalized to *β*-actin/GAPDH.

### 2.7. RNA Interference

The A549 cells were transfected with CD36 (sc-29995, Santa Cruz) or non-targeting siRNA for 24 h using INTERFER in siRNA transfection reagent (polyplus, jetPRIME, 101000027) according to the manufacturer’s guidelines, and then were used for further experiments.

### 2.8. Statistical Analysis

Statistical analysis was conducted using GraphPad Prism version 8.0. The results were presented as means ± SD and were analyzed using either one-way or two-way ANOVA, with subsequent appropriate post hoc tests to compare the means. *p* < 0.05 was considered a statistically significant difference.

## 3. Results

### 3.1. LA-m Potentiates Multiple Types of Cancer Cells to RSL3-Induced Ferroptosis

**Figure 1:**
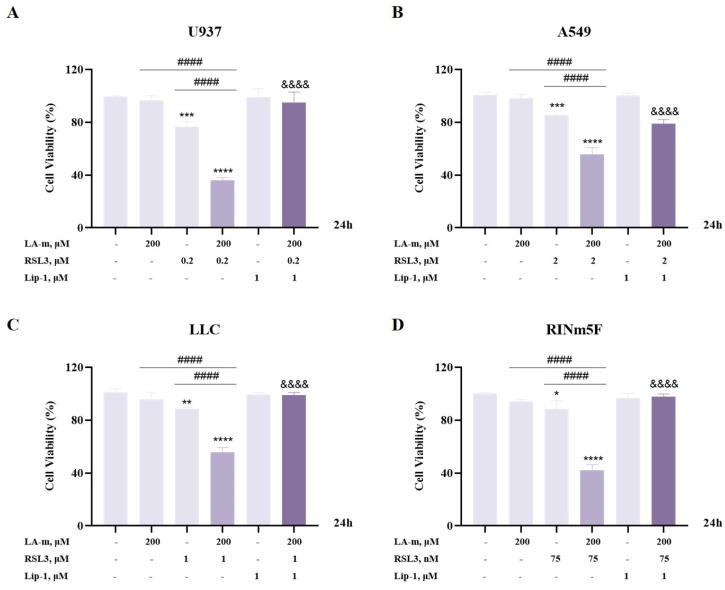
LA-m sensitized RSL3-induced ferroptosis in multiple types of cancer cells. (**A**) U937 human leukemia cells were treated with LA-m, RSL3 or their combination in the absence or presence of ferroptosis inhibitor Lip-1 for 24 h, and their viability was assayed using CCK8. (**B**–**D**) A549 lung cancer cells (**B**), Lewis lung cancer (LLC) cells (**C**), and RINm5F rat insulinoma cells (**D**) were exposed to LA-m and RSL3 alone or their combination in the absence or presence of ferroptosis inhibitor Lip-1 for 24 h, and the changes of cell viability were measured through crystal violet staining. Results are displayed as the mean ± SD (n = 3), * *p* < 0.05, ** *p* < 0.01, *** *p* < 0.001 and **** *p* < 0.0001 versus the control group. ^####^ *p* < 0.0001 versus the LA-m or RSL3 group. ^&&&&^ *p* < 0.0001 versus the combination group.

To assess the impact of medium-chain fatty acids on ferroptosis, U937 human leukemia cells, A549 lung cancer cells, LLC cells, and RINm5F rat insulinoma cells were treated with LA-m, RSL3, or their combination for 24 h, and the changes of cell viability were measured by CCK8 (for U937) or crystal violet staining. As shown in Figure 1, when compared with the untreated group, LA-m treatment alone did not affect the cell viability of any of the cell lines, and RSL3 treatment resulted in significant reductions in cell viability of varying degrees for all cell lines. However, whether compared with the control group or the LA-m or RSL3 treatment groups alone, the combination would result in a further significant reduction in cell viability, with U937 cell viability reduced to 36% (Figure 1A), A549 and LLC cell viability reduced to 55.9% (Figure 1B,C), and RINm5F cell viability reduced to 42.2% (Figure 1D). Thus, LA-m sensitized RSL3-induced cancer cell death.

To assess whether ferroptosis was involved in the enhanced reduction of cell viability, we examined the effect of a ferroptosis inhibitor on cell viability, with the results shown in Figure 1A–D. Treatment with the ferroptosis inhibitor Lip-1 alone showed comparable cell viability to the untreated group for all cell lines. Next, administration of Lip-1 in combination with LA-m and RSL3 significantly reversed the combination-induced decrease in cell viability, suggesting that LA-m potentiated RSL3-induced ferroptosis in multiple types of cancer cells.

### 3.2. LA-m Enhances the Cytotoxic Effect of LA-l on Cancer Cells

**Figure 2:**
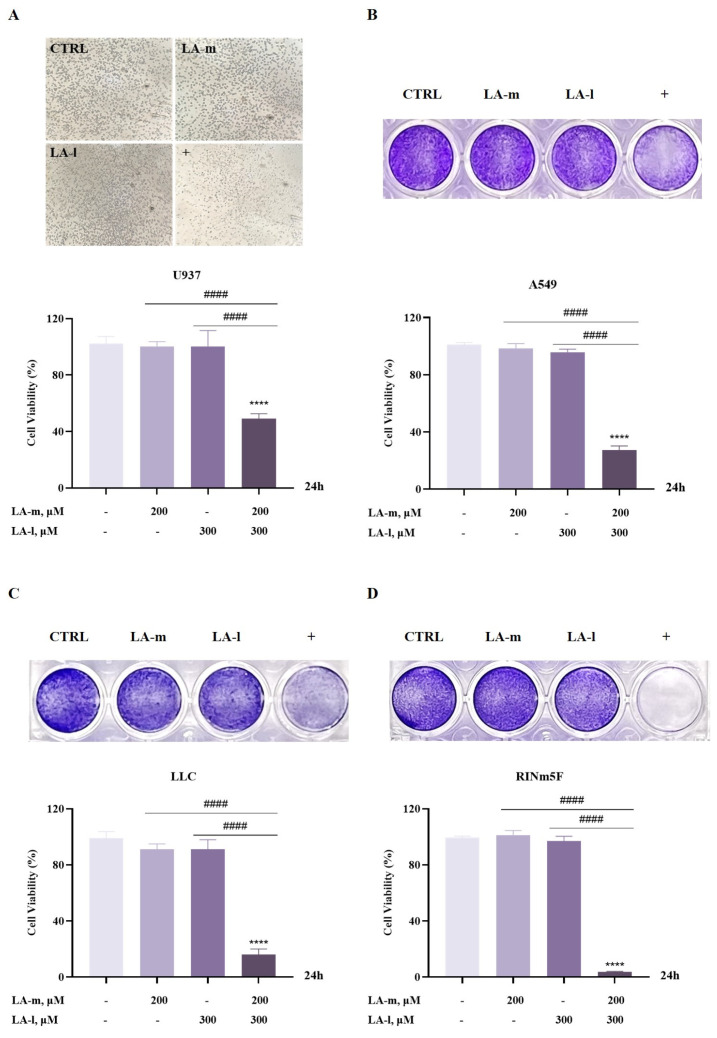
LA-m enhanced the cytotoxic effect of LA-l on multiple types of cancer cells. (**A**) U937 human leukemia cells were treated with LA-m, LA-l or their combination for 24 h, and cell viability was assessed by CCK8. (**B**–**D**) A549 lung cancer cells (**B**), Lewis lung cancer (LLC) cells (**C**), and RINm5F rat insulinoma cells (**D**) were treated with LA-m, LA-l or their combination for 24 h, and cell viability was determined by crystal violet staining. Results are represented as the mean ± SD (n = 3), **** *p* < 0.0001 versus the control group. ^####^ *p* < 0.0001 versus the LA-m or LA-l group. “+” means: combination of LA-m and LA-l.

It has been shown that ω-6 PUFAs, such as LA-l and AA, were able to induce ferroptosis in certain types of cancer cells [15,21,22,23,24,25], we then questioned whether the enhanced ferroptosis induction by LA-m and RSL3 combination could also be induced by combining LA-m with LA-l. Therefore, U937 human leukemia cells, A549 lung cancer cells, Lewis lung cancer (LLC) cells, and RINm5F rat insulinoma cells were employed to explore the combined cytotoxic effects of LA-m and LA-l at the same dose over 24 h. As shown in Figure 2, LA-m and LA-l alone were not significantly toxic to cell lines compared with the control group. CCK8 results showed that the combination of LA-m and LA-l reduced U937 cell viability to 49% (Figure 2A). The crystal violet staining results show that co-administration reduced A549 cell viability to 27.2% (Figure 2B), LLC cell viability to 16.1% (Figure 2C), and RINm5F cell viability to 3.6% (Figure 2D). In conclusion, LA-m enhanced the cytotoxic effect of LA-l on the cancer cells tested.

### 3.3. Ferroptosis Is Involved in the Enhanced Cytotoxic Effect of LA-m and LA-l Combination

**Figure 3:**
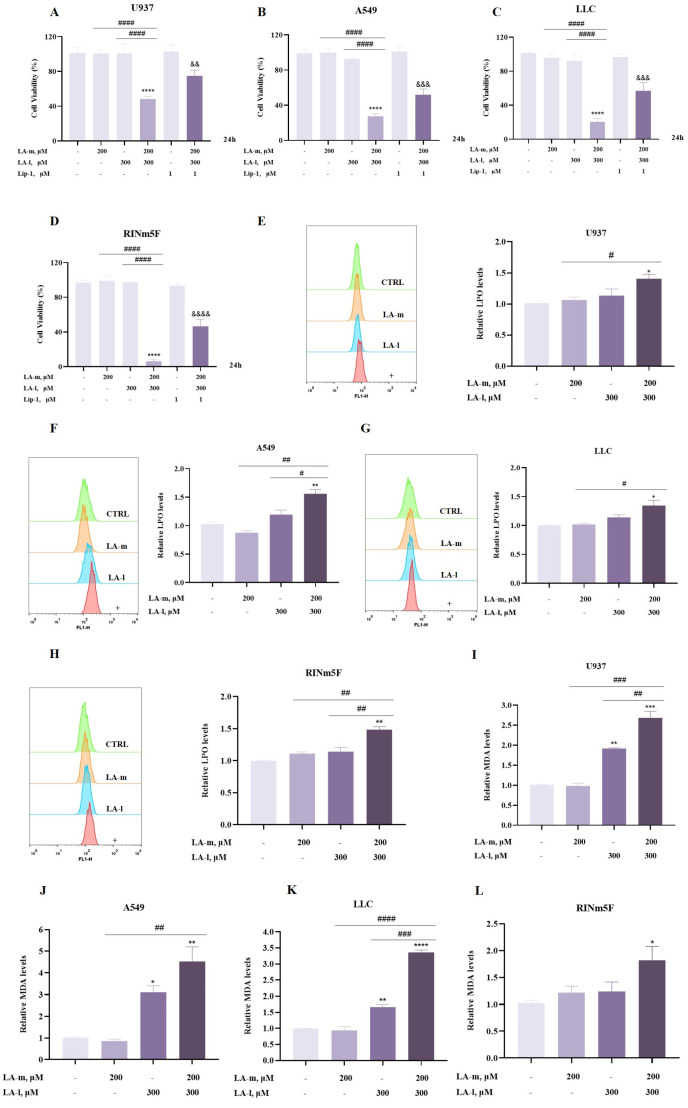
The enhanced cytotoxic effect induced by the combination of LA-m and LA-l is linked to ferroptosis. (**A**) U937 human leukemia cells were treated with LA-m, LA-l or their combination in the absence or presence of ferroptosis inhibitor Lip-1 for 24 h, and their viability was determined using CCK8. (**B**–**D**) A549 lung cancer cells (**B**), Lewis lung cancer (LLC) cells (**C**), and RINm5F rat insulinoma cells (**D**) were treated with LA-m and LA-l alone or their combination in the absence or presence of ferroptosis inhibitor Lip-1 for 24 h, and the changes of cell viability were measured by crystal violet staining. (**E**–**H**) After the treatment, the levels of intracellular LPO were quantified using flow cytometry after staining with Liperfluo in U937 human leukemia cells (**E**), A549 lung cancer cells (**F**), Lewis lung cancer (LLC) cells (**G**), and RINm5F rat insulinoma cells (**H**); “+” means: combination of LA-m and LA-l. (**I**–**L**) The content of intracellular MDA was measured by commercial kit in U937 human leukemia cells (**I**), A549 lung cancer cells (**J**), Lewis lung cancer (LLC) cells (**K**), and RINm5F rat insulinoma cells (**L**). Results are expressed as the mean ± SD (n = 3), * *p* < 0.05, ** *p* < 0.01, *** *p* < 0.001 and **** *p* < 0.0001 versus the control group. ^#^ *p* < 0.05, ^##^ *p* < 0.01, ^###^ *p* < 0.001 and ^####^ *p* < 0.0001 versus the LA-m or LA-l group. ^&&^ *p* < 0.01, ^&&&^ *p* < 0.001 and ^&&&&^ *p* < 0.0001 versus the combination group.

To determine whether ferroptosis participated in the enhanced decrease in cell viability induced by the combination of LA-m and LA-l, we investigated the effect of a ferroptosis inhibitor on cell viability, as well as on the levels of LPO and MDA, which are markers associated with ferroptosis. These results are presented in Figure 3. Treatment of a non-toxic dose of the ferroptosis inhibitor Lip-1 in U937, A549, LLC, and RINm5F cell lines partially rescued the decrease in cell viability induced by the combination of LA-m and LA-l (Figure 3A–D). Next, we determined the effect of co-treatment on LPO levels in each of the four cell lines, with the results shown in Figure 3E–H. Compared with the untreated or alone group, the co-treatments all resulted in a significant elevation of intracellular LPO levels. As further evidence of the induction of lipid peroxidation, MDA measurement by a commercial kit indicated that their combination substantially promoted LA-l-induced MDA generation (Figure 3I–L). Altogether, the results indicate that the cytotoxic effect enhanced by the combination of LA-m and LA-l is linked to ferroptosis.

### 3.4. The Sensitization Effect of LA-m on Ferroptosis Is Attributed to the Upregulation of CD36 and ACSL4

**Figure 4:**
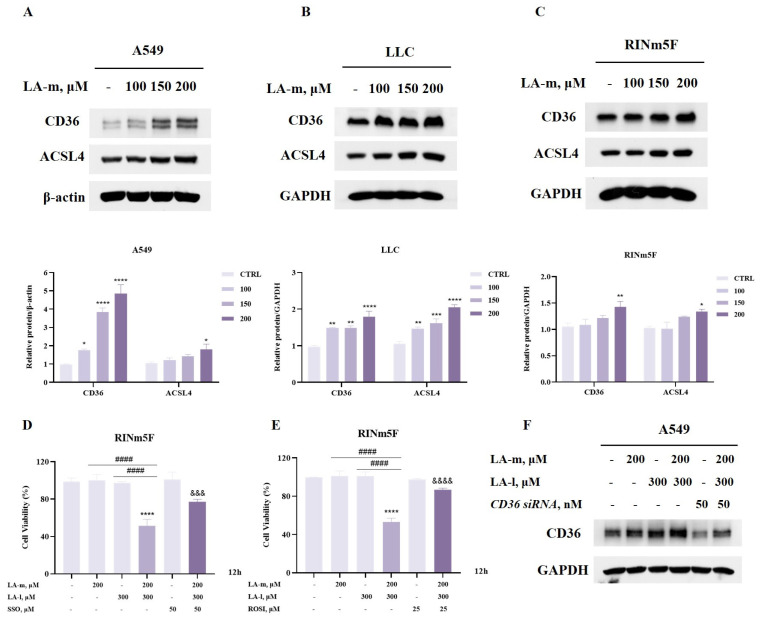
The potentiation effect of LA-m on ferroptosis is attributed to upregulating the CD36 and ACSL4 expression. (**A**–**C**) CD36 and ACSL4 protein levels in A549 lung cancer cells (**A**), Lewis lung cancer (LLC) cells (**B**), and RINm5F rat insulinoma cells (**C**) with treatment with LA-m for 12 h. (**D**,**E**) RINm5F rat insulinoma cells were treated with LA-m, LA-l or their combination in the absence or presence of SSO (**D**), a CD36 inhibitor, and ROSI (**E**), a ACSL4 inhibitor, for 12 h, and cell viability was assayed using the crystal violet staining. (**F**) The expression of CD36 in A549 cells after transfection with CD36 siRNA or negative-control siRNA. Results are expressed as the mean ± SD (n = 3), * *p* < 0.05, ** *p* < 0.01, *** *p* < 0.001 and **** *p* < 0.0001 versus the control group. ^####^ *p* < 0.0001 versus the LA-m or LA-l group. ^&&&^ *p* < 0.001 and ^&&&&^ *p* < 0.0001 versus the combination group.

CD36 is a key lipid transporter that is responsible for facilitating cellular long-chain fatty acid (such as LA-l) uptake across the plasma membrane, and is therefore involved in lipid metabolism-related ferroptosis sensitivity [26,27]. We hypothesized that the increased sensitivity of cancer cells to ferroptosis by LA-m might be associated with its upregulating effect on CD36 expression. The expression of CD36 was analyzed by Western blot and the results are presented in Figure 4A. The expression of CD36 was significantly upregulated following LA-m treatment in A549 cells, suggesting that LA-m might promote ferroptosis in cancer cells by increasing the uptake of long-chain fatty acids (LA-l). Similar results were also found in LLC cells (Figure 4B) and RINm5F cells (Figure 4C).

ACSL4 is an enzyme that is responsible for producing PUFA-derived acyl-CoAs, which is an essential step for lipid peroxidation during ferroptosis occurrence [28,29]. We speculated that induction of ACSL4 by LA-m might contribute to its ferroptosis sensitization. As shown in Figure 4A, treatment with LA-m resulted in a significant increase of ACSL4 levels in A549 cells. This result indicates that LA-m might facilitate the esterification of LA-l, thereby enhancing the sensitivity of cancer cells to ferroptosis. This finding is consistent in LLC cells (Figure 4B) and RINm5F cells (Figure 4C).

To determine the functional roles of CD36 and ACSL4 in LA-m-sensitized ferroptosis in multiple types of cancer cells, we assessed the effects of inhibiting CD36 and ACSL4 using the pharmacological inhibitors sulfosuccinimidyl oleate sodium (SSO) and rosiglitazone (ROSI) on ferroptosis; the results are presented in Figure 4D,E. The combination of LA-m and LA-l caused a significant reduction of cell viability in RINm5F cells. The decline in cell viability was partially reversed in the presence of SSO and ROSI, indicating that the upregulation of CD36 and ACSL4 by LA-m potentiated ferroptosis induction in RINm5F cells. In addition, the promoting effect of CD36 on LA-m-sensitized ferroptosis in A549 cells was further verified by a genetic approach, as shown in Figure 4F. This result shows that the specific siRNA targeting CD36 partially abolished the upregulated CD36 expression induced by the combination of LA-m and LA-l.

### 3.5. The Sensitization Effect Is Also Found with Medium-Chain Fatty Acids OA-m and DA-m

**Figure 5:**
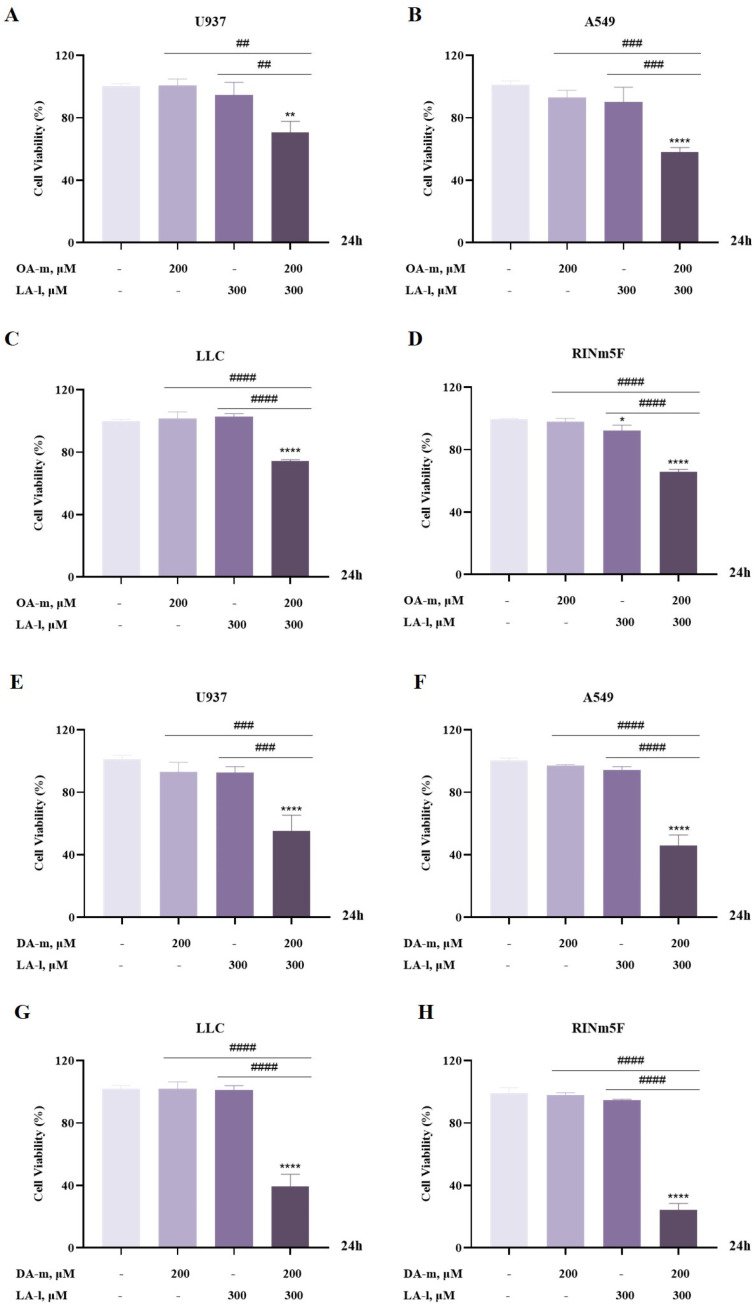
Medium-chain fatty acids OA-m and DA-m generally enhanced the cytotoxicity of LA-l on cancer cells. (**A**) U937 human leukemia cells were treated with OA-m, LA-l or their combination for 24 h, and their viability was assayed by CCK8. (**B**–**D**) A549 lung cancer cells (**B**), Lewis lung cancer (LLC) cells (**C**), and RINm5F rat insulinoma cells (**D**) were treated with OA-m, LA-l, or their combination for 24 h, and cell viability was evaluated by crystal violet staining. (**E**) CCK8 was used to determine the viability of U937 human leukemia cells induced by DA-m, LA-l, or their combination for 24 h. (**F**–**H**) Crystal violet staining was used to assess the viability of A549 lung cancer cells (**F**), Lewis lung cancer (LLC) cells (**G**), and RINm5F rat insulinoma cells (**H**) induced by DA-m, LA-l, or their combination for 24 h. Results are expressed as the mean ± SD (n = 3), * *p* < 0.05, ** *p* < 0.01 and **** *p* < 0.0001 versus the control group. ^##^ *p* < 0.01, ^###^ *p* < 0.001 and ^####^ *p* < 0.0001 versus the OA-m, DA-m or LA-l group.

The above experimental results have demonstrated that LA-m enhanced the cytotoxic effect of LA-l on cancer cells, and then we questioned whether the enhanced cell death induction by LA-m and LA-l combination could also be induced by combining other medium-chain fatty acids, such as octanoic acid (OA-m) or decanoic acid (DA-m), with LA-l. Next, we explored the combined cytotoxicity of OA-m and DA-m with LA-l on U937 human leukemia cells, A549 lung cancer cells, Lewis lung cancer (LLC) cells, and RINm5F rat insulinoma cells, and the results are shown in Figure 5. Compared with the untreated group, OA-m, DA-m or LA-l alone did not significantly affect cell viability. However, the combination of OA-m with LA-l decreased U937 cell viability to 70.7% (Figure 5A), A549 cell viability to 58.2% (Figure 5B), LLC cell viability to 74.1% (Figure 5C) and RINm5F cell viability to 65.9% (Figure 5D). Similarly, the combination of DA-m with LA-l decreased U937 cell viability to 55.3% (Figure 5E), A549 cell viability to 45.7% (Figure 5F), LLC cell viability to 39.5% (Figure 5G) and RINm5F cell viability to 24.3% (Figure 5H). Furthermore, we also found that DA-m enhanced the cytotoxicity of LA-l against cancer cells more than OA-m, and we speculated that this may be related to the length of the fatty acid carbon chain. In summary, medium-chain fatty acids generally enhanced the cytotoxicity of LA-l on cancer cells.

### 3.6. The Sensitization Effect Is Not Induced in Normal Cells

**Figure 6:**
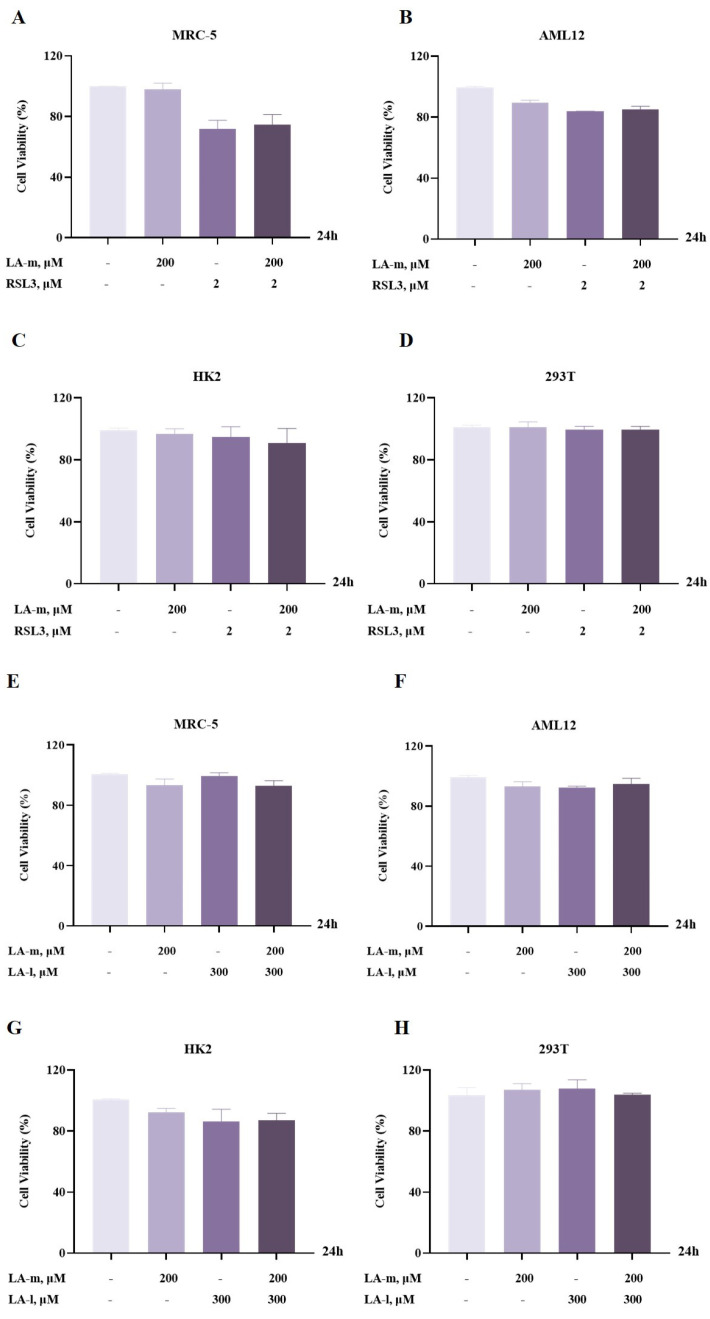
LA-m did not affect the viability of normal cells induced by RSL3 or LA-l. (**A**–**D**) MRC-5 human embryonic lung cells (**A**), AML12 alpha mouse liver cells (**B**), HK2 human proximal tubular epithelial cells (**C**), and 293T human embryonic kidney cells (**D**) were treated with LA-m, RSL3, or their combination for 24 h, and cell viability was assayed by crystal violet staining. (**E**–**H**) MRC-5 human embryonic lung cells (**E**), AML12 alpha mouse liver cells (**F**), HK2 human proximal tubular epithelial cells (**G**), and 293T human embryonic kidney cells (**H**) were treated with LA-m, LA-l, or their combination for 24 h, and the changes of cell viability were measured by crystal violet staining.

Many studies have shown that fatty acid regulation of ferroptosis is not only dependent on fatty acid types but is also closely related to cell types, such as cancer and normal cells. Our results indicate that LA-m significantly enhanced the sensitivity of multiple types of cancer cells to LA-l and RSL3-induced ferroptosis. To this end, we investigated the effect of LA-m on the sensitivity of normal cells to ferroptosis. The results show that LA-m did not further reduce the cell viability of normal cells, including MRC-5 human embryonic lung cells, AML12 alpha mouse liver 12 cells, HK2 human proximal tubular epithelial cells, and 293T human embryonic kidney 293 cells, induced by the classical ferroptosis inducer RSL3 (Figure 6A–D). In line with this, LA-m did not affect the cell viability of normal cells induced by LA-l (Figure 6E–H). Thus, we conclude that medium-chain fatty acids selectively increase the sensitivity of cancer cells to ferroptosis.

## 4. Discussion

As mentioned above, inducing ferroptosis in cancer cells is a promising strategy, particularly for therapy-resistant cancers. Increasing attention has been paid to identifying novel inducers or sensitizers of cancer cell ferroptosis. To this end, we found that medium-chain fatty acid LA-m significantly potentiated multiple types of cancer cells to RSL3 or LA-l-induced ferroptosis without increasing the toxic effect on normal cells, indicating that LA-m selectively enhances the sensitivity of cancer cells to ferroptosis induction. Furthermore, the sensitization effect was also found with other two types of medium-chain fatty acids, such as OA-m and DA-m, suggesting that such sensitization to ferroptosis is general application for medium-chain fatty acids. The findings of the present study clearly support the idea that medium-chain fatty acids hold potential to be developed as novel sensitizers of cancer cell ferroptosis so as to improve the efficacy of ferroptosis-based therapy.

Dysregulated iron metabolism-mediated accumulation of labile iron, iron-dependent lipid peroxidation of plasma membrane and the collapse of redox homeostasis are recognized as the key biochemical events during ferroptosis induction [4,30]. Among these, lipid peroxidation of plasma membrane not only serves as a hallmark of ferroptosis, but also as its executioner [12]. AA, a major metabolite of LA-l, serves as a major substrate for membrane lipid peroxidation during induction of ferroptosis. It is not surprising that LA-l or AA either endogenously or exogenously exposure has been repotted to exert promoting effect on ferroptosis. For example, Lee et al. demonstrated that supplementation with AA resulted in ferroptosis sensitization in gastric cancer cells [23]. Consistent with this, a study by Dierge et al. reported that excess uptake of ω-6 PUFAs, including docosapentaenoate (DPA), arachidonate (ARA) and linoleate (LA), selectively induce cytotoxic effects on acidic colon cancer cells through the induction of ferroptosis [15]. These findings support the idea that ω-6 PUFAs could be used for ferroptosis-mediated cancer therapy. Cellular uptake of ω-6 PUFAs is primarily mediated by membrane transport proteins, including fatty acid translocase (FAT/CD36), and CD36-mediated uptake of fatty acids plays a critical role in ω-6 PUFA-induced ferroptosis. Upregulation of CD36 is supposed to increase cellular uptake of ω-6 PUFAs, leading to elevated levels of ω-6 PUFAs, which in turn promotes ferroptosis induction. We then analyzed the influence of LA-m on the expression of CD36 to determine whether the sensitization effect of LA-m on LA-l-induced ferroptosis is associated with the upregulation of CD36 expression. As we expected, CD36 expression was significantly increased by LA-m, and blocking CD36 by its inhibitor and gene knockdown led to a reduction of the ferroptosis that is induced by the combination of LA-m and LA-l (Figure 4A–D,F), supporting our hypothesis.

A number of enzymes, including Acyl-CoA synthetase long-chain family member 4 (ACSL4), have been identified as being involved in lipid peroxide generation. In the process of lipid peroxidation, ACSL4 catalyzes the conversion of polyunsaturated fatty acids (PUFAs)—especially arachidonic acid (C20:4) and adrenic acid (C22:4)—to their respective acyl-coenzyme A (acyl-CoA) forms, which are further transformed by lysophosphatidylcholine acyltransferase 3 (LPCAT3) to form AA/AdA-phosphatidylethanolamine (PE), followed by oxidation via specialized enzymes lipoxygenase (ALOX) and cytochrome P450 oxidoreductase (POR) to generate AA/AdA-PE-OOHs, leading to ferroptosis [31]. The accumulation of lipid peroxides has been recognized as a determinant of ferroptosis induction. Activation of ACSL4 promotes ferroptosis, whereas inactivation of ACSL4 suppresses ferroptosis [32], and ACSL4 dictates ferroptosis sensitivity by regulating lipid metabolism. We then further tested whether ACSL4 was induced by LA-m, which in turn contributed to the enhanced ferroptosis. The results show that treatment with LA-m caused a dose-dependently increased ACSL4 in all cell lines tested (Figure 4A–C). Moreover, inactivating ACSL4 by its inhibitor led to a significantly decreased cell death induction (Figure 4E). Taken together, LA-m-mediated sensitization of cancer cells to ferroptosis was attributed to its ability to upregulate CD36 and ACSL4. These data provide a possible mechanical support for enhanced ferroptosis induction, achieved by a combination of medium-chain fatty acids and ferroptosis inducers.

There are several limitations in the present study that need to be addressed in future study. The first is that in vivo validation has not been performed. Our present study showed that, at in vivo achievable concentrations, MCFAs were able to sensitize multiple types of cancer cells to ferroptosis. We therefore speculate that the sensitization effect could be achieved in vivo, something which needs to be determined in animal studies. In addition to the efficacy, animal study should also address the pharmacokinetics, toxicity, and bioavailability of medium-chain fatty acids in cancer treatment. The second limitation regards whether the additional mechanisms (e.g., GPX4 or SLC7A11 inhibition, lipid peroxidation regulators like ALOXs) involved in the sensitization effect remain unaddressed. The third is that it is unclear whether the observed effects are specific to MCFAs or if LCFAs also induce similar ferroptosis sensitization.

## 5. Conclusions

**Figure 7:**
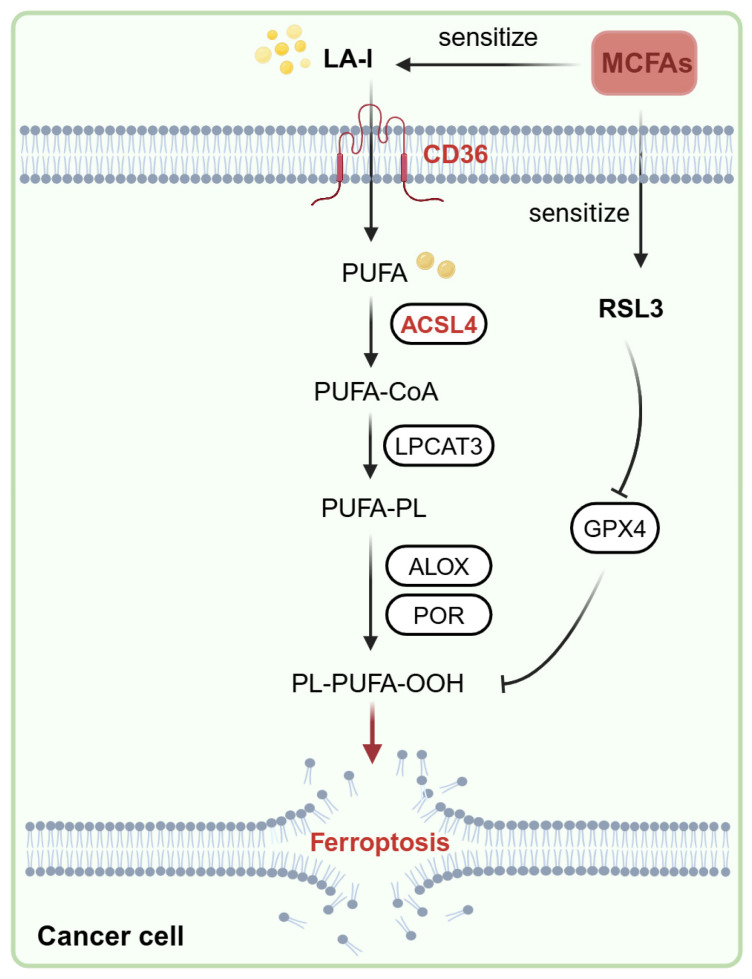
A schematic diagram of how medium-chain fatty acids (MCFAs) potentiate the sensitivity of cancer cells induced by RSL3 and selectively sensitize cancer cells to ferroptosis induced by linoleic acid (LA-l), which is mechanically associated with the upregulation of the expression of CD36 and ACSL4.

In summary, medium-chain fatty acids are capable of selectively sensitizing cancer cells to the ferroptosis that is mechanically associated with the induction of fatty acid transporter CD36 and lipid peroxidation related enzyme ACSL4 (Figure 7). These data suggest that medium-chain fatty acids could serve as selective ferroptosis sensitizers, enhancing the efficacy of ferroptosis-based cancer therapy.

## Data Availability

The original contributions presented in this study are included in the article. Further inquiries can be directed to the corresponding authors.
